# Endophenotypic correlates of cognitive function in reproductive-age individuals with polycystic ovary syndrome

**DOI:** 10.1016/j.xfre.2022.08.008

**Published:** 2022-09-06

**Authors:** Heather G. Huddleston, Kaitlin B. Casaletto, Eleni G. Jaswa, Natalie L. Rasgon, Pauline P. Maki, Marcelle I. Cedars, Lauri Pasch

**Affiliations:** a Department of Obstetrics, Gynecology and Reproductive Sciences, University of California San Francisco, San Francisco, California; b Memory and Aging Center, Department of Neurology, Weill Institute for Neurosciences, University of California San Francisco, San Francisco, California; c Department of Psychiatry and Behavioral Sciences, Stanford School of Medicine, Stanford, California; d Department of Psychology, University of Illinois Chicago, Chicago, Illinois; e Department of Psychiatry, University of California San Francisco, San Francisco, California

**Keywords:** Polycystic ovary syndrome, cognition, hyperandrogenism, depression, insulin resistance

## Abstract

**Objective:**

To characterize cognitive performance in relation to hormonal and metabolic factors in women with polycystic ovary syndrome (PCOS).

**Design:**

Cross-sectional study.

**Setting:**

Tertiary university center.

**Patient(s):**

A total of 48 individuals, aged 21–46 years, with PCOS according to the Rotterdam criteria.

**Intervention(s):**

Complete history and physical examinations, endovaginal ultrasounds, dermatologic assessments, neuropsychological assessments, and metabolic and hormonal serum tests.

**Main Outcome Measure(s):**

Sample-based z-scores on a comprehensive cognitive test battery.

**Result(s):**

Subjects were defined as having an androgenic (n = 31) or a nonandrogenic (n = 17) PCOS phenotype. Compared with their nonandrogenized counterparts, subjects with hyperandrogenism demonstrated lower relative performance on the tests of executive function (β-coefficient for the executive function composite z-score, −0.44; 95% confidence interval, −0.79 to −0.09), despite similar performance on the tests of memory, verbal reasoning, and perceptual reasoning. These differences were independent of age, years of education, and obesity. In an exploratory analysis in which subjects were stratified by the presence of insulin resistance (IR), subjects with PCOS with both IR and hyperandrogenism showed the lowest performance on a composite score of executive function, followed by those with hyperandrogenism alone.

**Conclusion(s):**

In this small study, subjects with hyperandrogenic PCOS demonstrated lower performance on the tests of executive function than subjects with nonandrogenic PCOS. Additional research is needed to confirm these findings in larger cohorts and investigate the role of modifiable factors, including IR, on cognitive outcomes.

Polycystic ovary syndrome (PCOS) is the most common endocrinopathy in females, diagnosed in up to 8% of women. Although defined by the presence of oligomenorrhea, hyperandrogenism, and/or polycystic ovaries (PCOs), Polycystic ovary syndrome also has metabolic manifestations, particularly intrinsic insulin resistance (IR), which occurs in up to 70% of patients (1, 2). In studies of non-PCOS populations, it is increasingly clear that a nexus exists between IR and neuropsychological outcomes, such as cognition performance, cognitive aging, and depression (3, 4, 5, 6, 7, 8, 9, 10). The underlying mechanisms are likely complex and may relate to altered insulin signaling in the brain and/or factors that are associated with systemic IR, such as inflammation (11). Similarly, the gonadal hormone levels can impact cognitive function and mood, although the role played by *female hyper*androgenism in relation to these outcomes has not been well described (12, 13).

Given that PCOS is defined by perturbations in both insulin sensitivity and gonadal hormone production, it is surprising that the relationship between PCOS and neuropsychological outcomes has only recently begun to be explored. In the last decade, several investigations have suggested that depression is increased in PCOS, and we and other investigators have uncovered associations between metabolic and hormonal markers and mood symptoms in this population (14, 15, 16, 17, 18, 19). There have been fewer studies of cognitive function, although 2 recent pilot studies in PCOS described subtle decrements in cognitive performance as well as differences in brain imaging measures (20, 21). These investigations raised the question of whether discrete PCOS features, many of which are modifiable, may contribute to decrements in cognitive performance in particular domains. Because cognitive performance deficits can impact the quality of life and professional attainment and may foretell adverse cognitive aging, there is a clear need for additional studies that better define neuropsychological outcomes in PCOS. Accordingly, the present study was designed to characterize cognitive performance in a sample of well-characterized subjects with PCOS and test the hypothesis that performance in specific domains would differ between individuals with hyperandrogenic PCOS relative and those with a nonandrogenic PCOS phenotype.

## Materials and methods

The study was approved by the University of California San Francisco (UCSF) Institutional Review Board. Written informed consent was obtained before initiating study procedures.

### Subjects

Study participation was offered to sequential individuals who sought an evaluation at a multidisciplinary clinic for PCOS between October 2017 and December 2018 (22). Evaluations were conducted according to a standard protocol described previously (23). Study inclusion required a diagnosis of PCOS by Rotterdam, defined by 2 of the following 3 parameters: hyperandrogenism, defined by either serum androgen levels above the normal range on screening laboratories and/or clinically significant hirsutism (modified Ferriman-Gallwey [mFG] score of ≥8); oligomenorrhea, defined as <8 cycles per year; and PCOs, defined as >12 follicles or a volume of >10 mL in an ovary. The mFG scores were determined by an examination by a board-certified dermatologist. The ovarian follicle criteria (>12) represent the guidance at the time of study launch (2017), before publication of the 2018 International Guidelines for PCOS recommending a higher threshold for follicle number (>20) (24). All subjects had discontinued hormone-suppressive medications at the time of initial PCOS evaluation, including oral contraceptives and spironolactone; however, some subjects had reinitiated medications at the time of the neuropsychological testing. Within the larger Rotterdam PCOS group, we further identified those with a hyperandrogenic PCOS phenotype (National Institutes of Health PCOS), defined as the presence of both oligo-ovulation *and* hyperandrogenism (n = 35) (25). The subjects who met the PCOS criteria *only* on the basis of oligomenorrhea and PCOs were designated as having PCOS without hyperandrogenism (n = 13). The exclusion criteria included age of <21 or >49 years, menopausal status, non-English fluency, pregnancy, breastfeeding, and/or history of organic brain injury.

### Evaluations

The subjects completed standardized questionnaires to collect detailed demographic and medical history information. Because the study procedures may have occurred several months after the initial PCOS evaluation, some subjects had resumed oral contraceptives at the time of neuropsychological and physiologic testing.

### Neuropsychological Testing

All subjects completed a thorough and standardized neuropsychological battery. Test administration and scoring were performed by trained personnel at the Center for Reproductive Health at the UCSF. Verbal reasoning and perceptual reasoning were measured using the Wechsler Adult Intelligence Scale (WAIS) (26). Memory was measured using the California Verbal Learning Trials long delay recall test (27). Processing speed was measured by WAIS symbol search. For an assessment of executive functions, we evaluated cognitive control, generativity, and working memory. In particular, the Delis-Kaplan Executive Function System was used to assess cognitive control (Stroop and Trail Making Tests) and generativity (Design and Verbal Fluency Test) (28). The WAIS Digit Span Test and Wechsler Memory Scale Symbol Span Test were used to assess working memory (29). Sample-based z-scores were calculated for cognitive outcomes. The Beck Depression Inventory, Second Edition, was self-administered to measure symptoms of depression, with a Beck Depression Inventory score of ≥20 considered as the threshold for moderate to severe symptoms (30).

### Physiologic Testing

The subjects also underwent morphometric and vital sign assessments and completed a 75-g glucose challenge with serum testing at baseline (fasting) and 30, 60, 90, and 120 minutes after challenge. Fasting samples were used to analyze additional hormonal and metabolic measures. The serum lipid and thyroid-stimulating hormone levels were measured on the day of sampling at Quest using nonfrozen specimens. Additional samples were processed and frozen on site. Hormonal and metabolic serum levels were measured in batch analysis at the University of Virginia Ligand Assay and Analysis Core Laboratory at completion of the study. Five percent of all samples were run in duplicate for quality control. Methodology and performance characteristics for all University of Virginia Ligand assays are available online (31). The glucose level after an oral glucose tolerance test was used to calculate the Matsuda insulin sensitivity index (32, 33).

### Statistical Analyses

Subject characteristics are presented as means ± standard deviation (SD) for continuous variables and were compared using Student’s *t*-test or the Mann-Whitney *U* test as appropriate. Categorical variables were compared using the chi-square test. Cognitive test scores were analyzed as sample-based z-scores, calculated by subtracting each participant’s score from the sample mean and dividing by the SD. An executive function composite score was generated as the mean of all z-scores for the 6 executive function tests. The linear regression models tested the effect of group (PCOS with hyperandrogenism vs. PCOS without hyperandrogenism) on cognitive test z-scores, controlling for age, years of education, and race/ethnicity. For exploratory analyses, we constructed regression models testing the effect of PCOS group on executive function composite z-score, incorporating covariates identified a priori as having potential to effect cognitive testing results, including body mass index (BMI), IR, depression, and medication usage at the time of cognitive testing (oral contraceptives and/or metformin). All data were stored in a Research Electronic Data Capture database and analyzed using Stata 14 (StataCorp, College Station, TX).

## Results

### Subject Characteristics

Forty-eight subjects between the ages of 21 and 46 years with PCOS defined by the Rotterdam criteria participated in the study. The mean (SD) age and BMI for all subjects were 31.0 ± 5.6 years and 32.3 ± 9.9 kg/m^2^, respectively. All subjects had private health insurance. The mean year of education was 16.6 ± 1.7. Subjects were stratified according to the presence or absence hyperandrogenism. Thirty-five subjects were defined as having PCOS with hyperandrogenism, whereas 13 were defined as having PCOS without hyperandrogenism. Group characteristics are shown in Table 1. Compared with subjects with PCOS without hyperandrogenism, those with PCOS with hyperandrogenism had slightly fewer years of education (mean, 17.3 vs. 16.2 years; *P* =.03) and higher triglyceride levels. By design, PCOS with hyperandrogenism demonstrated higher testosterone levels and mFG scores.

**TABLE 1:** Subject characteristics.

Characteristics	PCOS with hyperandrogenism (n = 35)	PCOS without hyperandrogenism (n = 13)	*P*
Age, y	30.3 (5.9)	32.3 (4.3)	.33
Education, y	16.2 (1.8)	17.4 (1.0)	.03
Ethnicity			
White Non-Hispanic	54.3%	76.9%	.46
Black	17.1%	7.7%	
Asian	5.7%	7.7%	
White Hispanic	22.9%	7.7%	
English first language			
No	18.2%	15.4%	.81
Yes	81.8%	84.6%	
Vital signs			
BMI, kg/m^2^	32.6 (9.8)	31.5 (10.4)	.58
Waist, inches	97.4 (23.88)	91.5 (17.6)	.26
Systolic blood pressure, mmHg	120.8 (11.84)	114.0 (10.7)	.04
Diastolic blood pressure, mmHg	70.1 (11.09)	69.1 (7.8)	.98
Reproductive measures			
mFG (hirsutism) score	12.73 (4.59)	5.15 (2.8)	<.0001
Total testosterone, (ng/dL)	38.7 (24.2)	20.8 (8.8)	.05
Sex hormone binding globulin, nmol/L	43.5 (6.9)	50.1 (4.6)	.56
Free androgen index	3.9 (2.4)	1.6 (0.9)	.01
Antimüllerian hormone, ng/mL	9.5 (4.2)	10.0 (1.1)	.3
Estradiol, pg/mL	120.1 (6.0)	106.1 (7.2)	.21
Metabolic measures			
Fasting glucose, mg/dL	88.92 (10.3)	83.82 (5.5)	.15
Fasting insulin, mIU/L	17.59 (16.5)	8.46 (8.1)	.08
Two-hour glucose, mg/dL	122.37 (35.5)	110.3 (30.2)	.38
HOMA-IR	4.2 (4.3)	1.82 (1.9)	.06
Abnormal HOMA-IR (>2.1)	15%	40%	.10
Matsuda index	5.53 (6.1)	6.82 (4.9)	.09
Cholesterol, mg/dL	189.81 (28.4)	182.85 (41.4)	.30
Triglycerides, mg/dL	116.85 (68.4)	85.92 (64.0)	.05
High-sensitivity C-reactive protein, mg/L	4.8 (4.6)	3.6 (4.2)	.40
TSH, mIU/L	1.41 (0.6)	1.7 (0.8)	.26
Psychological measures			
Depression score	16.1 (11.2)	14.1 (9.5)	.71
Moderate or severe depression			
No	65.7%	69.2%	.80
Yes	34.3%	30.8%	
Current medications			
Oral contraceptives	38%	36%	.51
Metformin	15%	12%	.52

*Note:* Data are presented as means (standard deviation) for continuous variables and compared with Student’s *t*-test or the Mann-Whitney *U* test as appropriate. Categorical variables were compared using the χ^2^test. Depression score was measured using the Beck Depression Index, with moderate to severe depression indicated by a score of ≥20. The mean values for testosterone, sex hormone binding globulin, free androgen index, and estradiol exclude subjects on oral contraceptives (n = 17) at the time of serum sampling. BMI = body mass index; HOMA-IR = Homeostatic Model Assessment for Insulin Resistance; mFG = modified Ferriman-Gallwey; PCOS = polycystic ovary syndrome; TSH = thyroid-stimulating hormone.

### Differences in Cognitive Function Between PCOS with Hyperandrogenism and PCOS Without Hyperandrogenism

To compare the effects of the PCOS group on cognitive function, we performed the linear regression models treating cognitive test z-scores as continuous outcomes, controlling for age, years of education, and race/ethnicity. The results are shown in Table 2. We observed no differences in the tests of verbal reasoning, perceptual reasoning, processing speed, and memory, indicators of premorbid intelligence quotient. However, within the executive function domain, we found that PCOS with hyperandrogenism was independently associated with decreased performance on the tests of cognitive control, generativity, and working memory as well as on an executive function composite z-score, which incorporated all 6 executive function tests (β-coefficient, −0.44; 95% confidence interval, −0.79 to −0.09; *P* =.016).

**TABLE 2:** Associations between the PCOS group and cognitive test z-scores.

Domain	Test	Coefficient (95% CI)	*P*
Verbal reasoning	WAIS vocabulary	−0.43 (−1.08 to 0.22)	.19
Perceptual reasoning	WAIS matrix	−0.41 (−1.08 to 0.26)	.22
Memory	CVLT long delay recall	0.02 (−0.65 to 0.70)	.94
Processing speed	WAIS symbol search	−0.55 (−1.7 to 0.07)	.08
Executive function			
Cognitive control	DKEFS Trail Making B	−0.64 (−1.27 to 0.0)	.05
	DKEFS Stroop Interference Test	−0.53 (−1.2 to 0.14)	.12
Generativity	DKEFS Design Fluency	−0.39 (−0.99 to 0.21)	.20
	DKEFS Verbal Fluency	−0.62 (−1.2 to −0.2)	.04
Working memory	WAIS Digit Span Backward	−.75 (−1.46 to −0.02)	.04
	WMS IV Symbol Span (visual)	−0.18 (−0.79 to 0.43)	.56
Composite executive function		−0.44 (−0.79 to −0.09)	.016

*Note:* The models tested the effect of PCOS with hyperandrogenism compared with PCOS without hyperandrogenism, controlling for age, years of education, and race/ethnicity. Composite executive function was the mean of z-scores for all 6 executive function tests. CI = confidence interval; CVLT = California Verbal Learning Trials; DKEFS = Delis-Kaplan Executive Function System; WAIS = Wechsler Adult Intelligence Scale, Fourth Edition; WMS = Wechsler Memory Scale, Fourth Edition.

We next constructed regression models testing the effect of the PCOS group on executive function composite z-score, incorporating covariates identified a priori as having the potential to impact cognitive testing results, including BMI, IR, depression, and medication usage at the time of testing (oral contraceptives and/or metformin) (Table 3). In all models, the significance of the effect of PCOS with hyperandrogenism on an executive function composite z-score was maintained (β-coefficient for the fully adjusted model, 0.41; 95% confidence interval, −0.74 to −0.08; *P*=.016). Of the covariates considered, only metformin usage was also associated with a significant negative effect.

**TABLE 3:** Associations between the characteristics and an executive function composite z-score.

Variable	β-coefficients		
	Model 1	Model 2	Model 3
PCOS with hyperandrogenism vs. PCOS without hyperandrogenism	−0.439 to −0.440	−0.391^a^	−0.410^a^
Age	−0.004	−0.004	−0.002
Education	0.046	0.049	0.057
BMI (kg/m^2^)		−0.005	−0.005
Insulin resistance		0.039	−0.014
Depression			−0.002
Metformin use			−0.418^a^
Oral contraceptive use			−0.010

*Note:* The executive function z-score was inclusive of all 6 executive function tests. Model 1, PCOS group, age, years of education, and ethnicity/race. Model 2, all variables from model 1, insulin sensitivity index (log of Matsuda insulin sensitivity index), and BMI (continuous). Model 3, all variables from model 2, depression symptom score (Beck Depression Inventory, Second Edition), and current use of metformin and/or oral contraceptive (categorical). In all models, PCOS with hyperandrogenism vs. without hyperandrogenism demonstrated a significant negative effect on executive function z-score (*P*<.01). BMI = body mass index; PCOS = polycystic ovary syndrome.

a *P* < .05.

**FIGURE 1:**
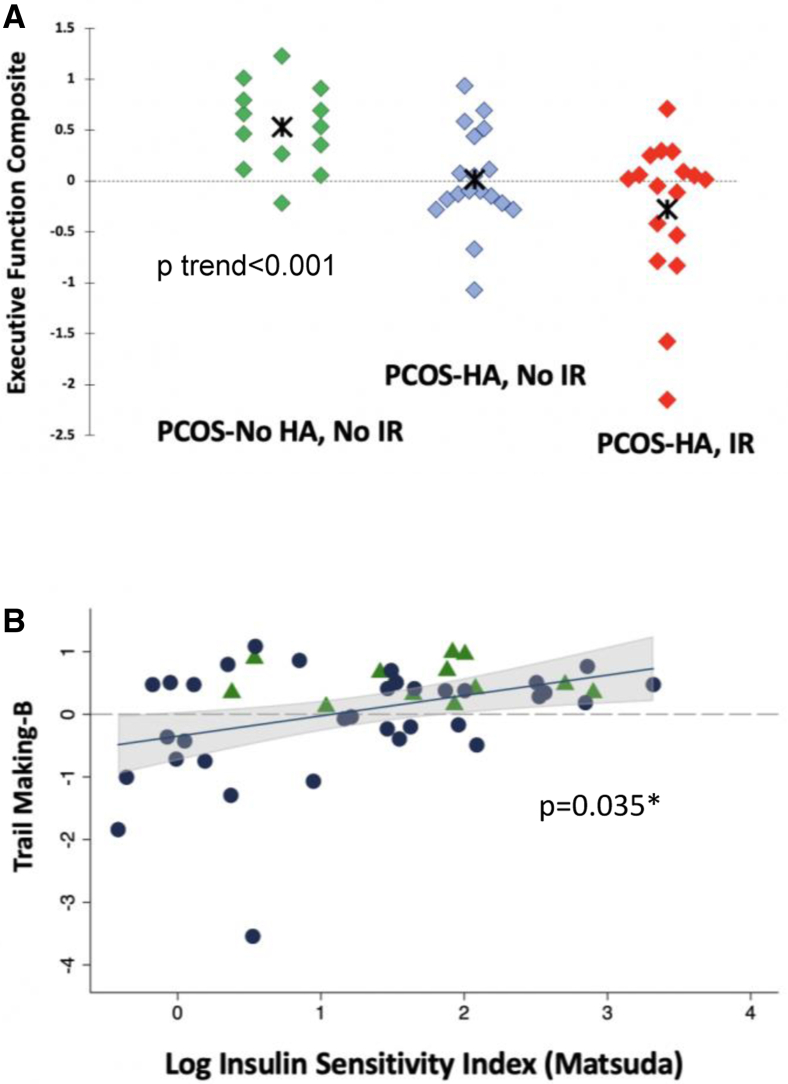
(A) Executive function composite z-scores for polycystic ovary syndrome (PCOS) without hyperandrogenism and without insulin resistance in green (PCOS-No HA, No IR), PCOS with hyperandrogenism and without insulin resistance in blue (PCOS-HA, No IR), and PCOS with hyperandrogenism and insulin resistance in red (PCOS-HA, IR). Insulin resistance defined as a Homeostatic Model Assessment for Insulin Resistance score of >2.1. Polycystic ovary syndrome without hyperandrogenism but with insulin resistance (n = 2) not included. The cross indicates means; *P* trend <.001. (B) Scatterplot of the Trail Making B z-scores and log insulin sensitivity index (Matsuda), with PCOS-HA (blue circles) and PCOS-No HA (green triangles) depicted. The shaded area shows 95% confidence interval for linear relationship; P value adjusted for age, years of education, and ethnicity/race.

To explore the role of IR on group outcomes, we stratified by presence or absence of clinically significant IR (Homeostatic Model Assessment for Insulin Resistance score of >2.1). Considering subjects with PCOS without hyperandrogenism and without IR as the reference group, we found that hyperandrogenic subjects with PCOS both with (n = 18) and without (n = 17) IR had lower executive function composite z-scores, with the lowest scores for those with IR (nonparametric test for trend *P*=.001) (Fig. 1A). We also investigated the potential linear relationships between IR and executive function outcomes. On an exemplar test of cognitive control (Trail Making B), we found evidence of a linear relationship between insulin sensitivity (log Matsuda insulin sensitivity index) and cognitive control performance, controlling for age, years of education, and ethnicity (Fig. 1B).

## Discussion

In this study, we found that a group of individuals with hyperandrogenic PCOS demonstrated similar performance on the tests of memory and reasoning compared with a control group of individuals with nonandrogenic PCOS, suggesting similar premorbid intelligence quotient. In contrast, we found significantly lower performance on tests within the executive function domain, such as cognitive control, for those with the hyperandrogenic phenotype. Executive functions encompass a broad set of cognitive processes that are needed to select and monitor behaviors in pursuit of a goal. Deficits in executive functioning can impair one’s ability to organize tasks and control behaviors, leading to challenges in both personal and occupational realms (34). The observed relationship between hyperandrogenic PCOS and executive function performance was independent of demographic factors and mood symptoms, suggesting that the endophenotypic features of PCOS could impact cognitive processes.

An emerging body of work has identified brain health as an area of concern for individuals with PCOS. In the psychological domain, a heightened risk of depression and anxiety in PCOS has been well established (14, 16). However, whether individuals with PCOS have differences in cognitive functioning has been only minimally investigated. Indeed, our report adds to a small body of emerging work on cognitive health in this population. In 2007, Schattman et al. (35) found a lower score on the tests of verbal fluency, verbal memory, and manual dexterity in a group of subjects with PCOS (n = 29) than that in age-matched controls. More recently, several small studies have suggested differences in cognitive performance and brain imaging findings in PCOS (20, 21, 36). We sought to both extend these investigations and investigate potential endophenotypic correlates of cognitive outcomes. Our finding of lower cognitive performance in the executive function domain for those with a classic, androgenic PCOS than for those with nonandrogenic PCOS begins to delineate a potential role for hyperandrogenism in mediating cognitive functions in this population.

Currently, little is known about how hyperandrogenism may influence cognition in reproductive-age women. The sexual differentiation of the brain is programmed by sex steroid secretion at various periods during development. This programming is thought to result in subtle differences in particular cognitive domains, such as increased spatial awareness in males and increased verbal functions in females (13, 37). It is possible that our finding of lower verbal fluency in those with hyperandrogenic PCOS is reflective of the influence of increased androgens during development, either in utero or at puberty. However, we also observed differences in cognitive control and working memory, which had not been shown to be sexually differentiated, suggesting that other factors correlated with hyperandrogenism may actually underlie these observations (38).

We also found evidence that IR may serve as a determinant of executive function performance in PCOS. This finding was not entirely surprising because IR has been linked to poor executive functioning in younger populations and predicts adverse neurobehavioral outcomes with aging (3, 4, 5, 6, 7, 8, 9, 10, 39, 40, 41, 42). Although the mechanisms are unclear, recent work has focused on altered insulin signaling in the brain (central IR), wherein insulin receptors in key brain areas may be resistant to the actions of insulin (11, 43). Additionally, factors associated with, or resulting from, IR, such as hyperglycemia, inflammation, obesity, and/or vascular damage, may play a role in mediating adverse neurobehavioral outcomes, particularly in aging populations (44, 45, 46, 47). Taken together, our findings raise the possibility that a combination of both hormonal and metabolic factors may impact cognition in PCOS.

Several points should be considered when interpreting our results. For one, our findings should not be interpreted as indicating impairment in cognitive function in our groups. Instead, these analyses suggest only the presence of focal cognitive differences *in relation to* PCOS features. Moreover, the clinical importance of these differences, if any, will require further investigation. For example, the impact of executive function differences on health behaviors in PCOS should be explored. Executive function deficits have previously been linked to challenges in executing healthy behaviors, such as exercise or dietary interventions (48, 49). Because appropriate diet and exercise habits are a critical for supporting long-term health in PCOS, methods to understand and improve executive function skills may be a new tool to help those with PCOS achieve improved behaviors. Furthermore, if additional work determines that potentially treatable or manageable features of PCOS (e.g., hyperandrogenism or IR) contribute to executive function deficits, there may be opportunities to improve cognitive outcomes through early interventions to manage hormonal and/or metabolic abnormalities.

An important strength of this study is a design that compared well-matched subjects with classic hyperandrogenic PCOS with subjects with a mild PCOS phenotype, all recruited from the same PCOS center. This strategy allowed us to minimize selection bias. However, by comparing cognitive performance within 2 PCOS groups, our study may underestimate effects that would be observed when comparing to non-PCOS controls. Larger studies with well-matched non-PCOS controls are needed to better contextualize these findings and interrogate the role of additional PCOS factors, including menstrual disturbance. Several additional limitations should be considered. First, given the cross-sectional design of this study, we are unable to infer causality. Second, our sample size was small. Additional work is needed to confirm these findings with larger populations and further elucidate relationships between distinct endophenotypic features of PCOS and brain health. Finally, this study included only premenopausal women in the third, fourth, and fifth decades of life. Studies are needed to clarify the role of age and aging for people with PCOS.
